# Factors Associated With Insecticide‐Treated Bed Net Possession and Utilization in Malaria Prevention Among Fulani Pregnant Women in the Savannah Hinterlands of Ghana: A Cross‐Sectional Study

**DOI:** 10.1002/hsr2.72415

**Published:** 2026-04-20

**Authors:** Yula Salifu, Joseph Lasong, Eleonora Bakintewune Wobi, Torjim Salifu, Bismark Nantomah, Gordon Dandeebo

**Affiliations:** ^1^ Department of Population and Reproductive Health, School of Public Health University for Development Studies Tamale Ghana; ^2^ Department of Biomedical Laboratory Sciences, School of Allied Health Sciences University for Development Studies Tamale Ghana; ^3^ Department of Sociology and Social Work, Faculty of Social Science and Arts SD‐Dombo, University of Business and Integrated Development Studies, WA Bamahu Ghana

**Keywords:** Fulani, Ghana, ITN, malaria, possession, pregnancy, utilization, women

## Abstract

**Background and Aim:**

Malaria poses a major threat to pregnant women in Ghana, especially among marginalized nomadic Fulani communities. This study examined factors influencing insecticide‐treated net (ITN) ownership and use among Fulani pregnant women in northern Ghana.

**Methods:**

A cross‐sectional survey was carried out between April and June 2022 among Fulani pregnant women in the West Gonja Municipality. Recruitment combined a limited community census with peer‐assisted snowball sampling to capture this mobile population. Data were collected through structured, face‐to‐face interviews in local languages after obtaining informed consent. Multivariable binary logistic regression was applied to identify factors associated with ITN ownership and, among owners, predictors of utilization. Statistical significance was set at *p* ≤ 0.05.

**Results:**

Of 159 participants, 54.7% (*n* = 87) reported owning an ITN. Among owners, usage was 52.9% (*n* = 46/87). Predictors of ITN ownership included awareness that ITNs prevent malaria ([adjusted odds ratio, AOR] = 3.45, 95% [confidence interval, CI]: 1.33–8.94), prior counseling on ITN use (AOR = 4.52, 95% CI: 1.53–13.34), and lower wealth status (AOR = 0.21, 95% CI: 0.09–0.49). Utilization was linked to knowledge of malaria symptoms (AOR = 0.18, 95% CI: 0.05–0.64), having more than three children (AOR = 0.02, 95% CI: 0.002–0.12), and owning multiple ITNs (AOR = 0.07, 95% CI: 0.02–0.28).

**Conclusions:**

ITN ownership and use among Fulani pregnant women were far below national and global targets. Possession did not translate into consistent use. Strengthened health education and tailored, mobile‐responsive distribution strategies are essential to improve malaria prevention in this underserved population.

**Abbreviations**
ANC: antenatal care
FPW: Fulani pregnant woman
GHS: Ghana Health Service
GSS: Ghana Statistical Service
IPTp‐SP: Intermittent Preventive Treatment of Malaria with Sulphadoxine‐Pyrimethamine
ITN: Insecticide Treated Bed Net
LMICs: low‐middle‐income countries
PCA: principal components analysis
RBM: roll back malaria
SMC: seasonal malaria chemoprevention
SPSS: Statistical Package for Social Scientists
UDS: University for Development Studies
WHO: World Health Organization

## 1 Background

Malaria remains a leading cause of preventable illness and death, with the greatest burden in low‐ and middle‐income countries (LMICs) [1]. In pregnancy, malaria increases risks of maternal anemia, fetal growth restriction, low birth weight, and perinatal loss. The World Health Organization (WHO) recommends a package of malaria in pregnancy interventions especially insecticide‐treated nets (ITNs) and intermittent preventive treatment with sulfadoxine‐pyrimethamine (IPTp‐SP) to mitigate these harms [2]. A review showed that ITNs reduce malaria episodes and all‐cause child mortality in endemic settings and remain a cost‐effective cornerstone of prevention [3].

WHO policy targets universal access and use of proven vector‐control tools; achieving this requires delivery models that reach underserved groups as well as behavior change to sustain consistent ITN use [4]. Ghana continues to face substantial malaria transmission, with the northern savannah belt experiencing the most intense and seasonal transmission and a high share of outpatient and inpatient visits attributable to malaria [5]. While national distribution campaigns and continuous channels have expanded household access to ITNs, use has not kept pace with ownership [6, 7]. This ownership‐to‐use gap is well documented across Africa and is influenced by net condition, heat/discomfort, sleeping arrangements, and intra‐household allocation [8, 9].

Ghana‐based studies mirror these patterns among women and pregnant women. While social networks can shape ITN uptake [10], a persistent gap between ownership and use is reported across multiple regions in Ghana [11, 12, 13]. This highlights that access alone does not guarantee consistent use. A recent scoping review in Ghana synthesizes facilitators (e.g., antenatal care [ANC] distribution, risk awareness) and barriers (e.g., perceived discomfort, net hanging constraints, misconceptions), underscoring the need for tailored, context‐specific strategies to translate access into nightly protection [6].

Nomadic and pastoralist populations face additional structural barriers. Mobility, temporary shelters, outdoor sleeping, and peripheral settlement can lead to missed contacts with routine health services and mass campaigns, as well as practical challenges to hanging and maintaining nets [14, 15]. Mixed methods studies among Arab, Dazagada and Fulani pastoral nomads in Chad documents knowledge gaps, social beliefs, and practices that influence long‐lasting insecticidal net (LLIN) ownership and use [14]. In northern Senegal, nomadic pastoralists report prevention and care‐seeking constraints that further depress effective coverage [15]. These findings are directly relevant to Ghana's Fulani communities in the northern savannah, whose livelihoods depend on seasonal mobility.

Gender and household decision‐making are also critical, with evidence from Ghana showing men frequently influence key maternal health decisions [16]. This is particularly relevant for nomadic Fulani communities, where patriarchal structures and mobility may uniquely shape women's access to and use of preventive resources like ITNs. Yet most Ghanaian ITN studies either aggregate pregnant women without disaggregating nomadic Fulani, or they focus on settled communities [10, 11, 12, 13]. As a result, programmatic insights derived from sedentary populations may not capture the distinct constraints and facilitators that shape ITN possession and use in mobile pastoralist households.

To our knowledge, no prior study in Ghana has examined predictors of ITN possession and use specifically among nomadic Fulani pregnant women. This study addresses that gap by identifying individual, household, and community‐level factors associated with ITN ownership and actual utilization among Fulani pregnant women living in Ghana's northern savannah. In grounding analysis in documented barriers (mobility, settlement patterns, sleeping arrangements, and gendered decision‐making) and in Ghana's program experience, findings are intended to inform mobile LLIN delivery, integration with ANC/outreach, male‐engagement approaches, and culturally resonant communication to convert access into nightly protection [4, 5, 6, 10, 11, 12, 13, 14, 15, 16].

## 2 Methods and Materials

### 2.1 Study Area

**Figure 1:**
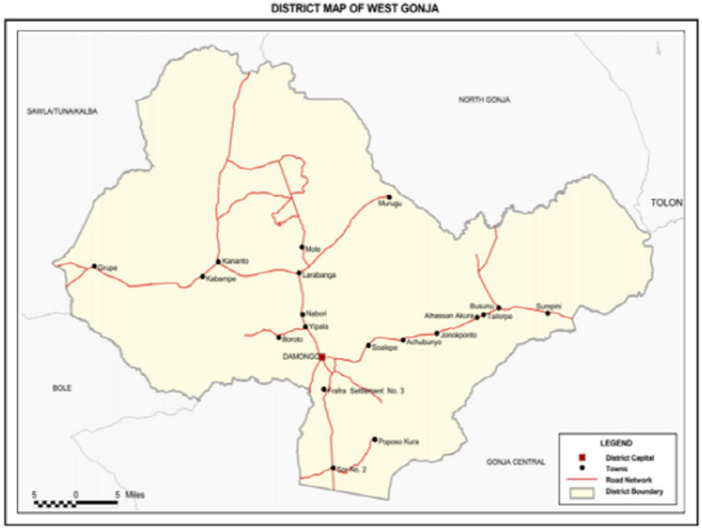
The map of the West Gonja Municipality. *Source:* Ghana Statistical Service, GIS.

The study was conducted in the West Gonja Municipality of the Savannah Region, Ghana. The municipality lies west of Tamale, the Northern Regional capital, between latitude 8°32′–10°21′ N and longitude 1°05′–2°58′ W, with Damongo serving as the administrative capital. It covers an estimated land area of 4700 km², of which about 30% is occupied by forest reserves and the Mole National Park [17]. The municipality has a population of approximately 53,700 people. The Gonja ethnic group is the majority, while minority groups include the Dagomba, Waala, Dagaaba, Frafra, Hausa, and Fulani [17]. Women of reproductive age are estimated at 12,882, with an expected 2173 pregnancies annually [17]. Health services are delivered through 26 facilities, though geographic spread and population mobility limit accessibility for nomadic groups such as the Fulani [18]. Climatic conditions in West Gonja are strongly linked to malaria transmission. The municipality experiences a bimodal rainfall pattern, averaging 1144 mm annually. Rainfall typically begins in late April and ends in October, with peak rains in June–July and a brief dry spell in August [17]. Intense downpours, sometimes exceeding 300 mm per hour, create stagnant pools and erosion‐prone areas, which provide breeding sites for malaria vectors [17]. The long dry season, from November to March, also contributes to population movements as nomadic Fulani migrate in search of pasture, potentially reducing their access to malaria prevention and control services. This ecological and demographic context makes West Gonja a high‐risk setting for malaria transmission, especially among pregnant Fulani women whose mobility, marginalization, and limited access to health facilities further constrain utilization of preventive interventions. Figure 1 depicts the map of the West Gonja Municipality.

### 2.2 Study Design, Population, and Sampling

This study employed a community‐based cross‐sectional design conducted between April and June 2022 among Fulani pregnant women residing in the West Gonja Municipality, Savannah Region of Ghana. Pregnant women are a key focus group in malaria control strategies due to their heightened vulnerability, alongside children under five. Eligible participants were self‐identified Fulani women confirmed pregnant and resident in the study area for at least 6 months. Women who declined participation, were critically ill, or unable to provide informed consent were excluded.

A total of 159 Fulani pregnant women were recruited. Estimating the Fulani population was challenging because they are highly mobile, sparsely distributed, and excluded from routine surveys, making them a recognized hard‐to‐reach group [19]. To overcome the absence of a sampling frame, a combination of census and snowball sampling was applied. In communities with known Fulani households, enumerators attempted a census by approaching all eligible women. Where this was not possible, snowball sampling was employed: initial “seeds” (women identified with the assistance of the West Gonja Municipal Health Directorate and local community partners) were purposively chosen from diverse Fulani sub‐groups to enhance heterogeneity. These seeds referred peers within their networks, who in turn referred others until recruitment was saturated.

To minimize selection bias, two respected community members facilitated introductions and rapport building with households, ensuring inclusion of less‐visible groups. This chain‐referral process has been widely used to access marginalized populations and has been shown to improve trust and participation among groups who may otherwise avoid formal enumeration [20].

Fieldwork was conducted in the Busunu and Canteen sub‐municipalities, which host the largest Fulani settlements. Ten of the 13 Fulani‐dominated communities were included, while three were excluded due to inaccessibility and poor road conditions. The combined census–snowball approach was designed to capture a diverse sample or aimed to reach a broad spectrum of Fulani pregnant women in the municipality, while addressing the unique challenges of working with a nomadic, socially marginalized population.

### 2.3 Data Collection and Quality Control

A structured questionnaire was developed following a detailed review of existing literature [21, 22, 23] and expert consultations. The tool captured demographic and maternal health variables, including ANC attendance, malaria knowledge, and possession and use of ITNs. Face‐to‐face interviews were conducted with pregnant women to ensure clarity and completeness of responses. The study questionnaire is provided as Supporting Information: File 1.

Household socioeconomic status was assessed using principal component analysis (PCA) of 21 household asset indicators encompassing productive, non‐productive, and housing characteristics (e.g., bicycle, motorcycle, mobile phone, mattress, source of drinking water, type of toilet facility, roofing structure, and livestock ownership) [24]. To improve validity, items with very high (> 95%) or very low (< 5%) prevalence were excluded, leaving nine indicators for PCA. Each household was assigned a wealth score, ranked, and classified into tertiles (poor, middle, and wealthy), in line with methods previously applied in ITN studies [22, 23].

Data collection was conducted by four trained enumerators who were fluent in Hausa/Fulfulde local Ghanaian languages, and English. Enumerators underwent a 2‐day training on research ethics, interview techniques, and data quality assurance. The questionnaire was translated into Hausa/Fulfulde and back translated into English to ensure semantic accuracy. Pretesting was conducted in a nearby community with 20 participants who share similar sociodemographic characteristics, which enabled refinement of question wording, flow, and inclusiveness.

Daily field supervision was carried out to monitor consistency, and questionnaires were reviewed at the end of each day for completeness. Respondents unable to answer independently were assisted, and interviews lasted between 20 and 45 min. To ensure methodological transparency and reporting rigor, the study adhered to the Strengthening the Reporting of Observational Studies in Epidemiology (STROBE) guidelines [25].

Ethical approval for the study was obtained from the Institutional Review Board of the University for Development Studies with reference ID: [UDS/RB/059/22]. Permission was also sought from community leaders prior to data collection. Written informed consent was obtained from each participant, and in cases of limited literacy, the consent form was read aloud in the respondent's preferred language, with thumbprints used to document consent. Confidentiality was maintained throughout the study, and participants were assured of the voluntary nature of their participation and their right to withdraw at any time without penalty.

### 2.4 Dependent Variables

This study has two main dependent variables: ITN possession and ITN utilization.

### 2.5 Operational Definitions

Household: Defined as a husband, wife/wives, and/or dependents (children, grandparents) living together and sharing meals from the same cooking pot.

ITN possession: A respondent was considered to possess an ITN if her household owned at least one insecticide‐treated net, whether long‐lasting or re‐treatable.

ITN utilization: Defined as having slept under an ITN the night before the survey. Analysis of utilization was restricted to those who possessed an ITN.

Knowledge of malaria: Measured through questions on causes, symptoms, prevention, and consequences. Scores ≥ the median (≥ 4) were classified as good knowledge.

Practice towards malaria prevention: Categorized as good if respondents both possessed an ITN and reported recent use, and poor if they did not possess or did not use one.

### 2.6 Statistical Analysis

Statistical Package for Social Scientists (SPSS) version 25.0 was used for the analysis. Descriptive statistics were produced for means, standard deviations, percentages, and frequencies. A bivariable analysis with chi‐square/Fishers' exact test of association was conducted to identify the factors associated with the outcome variables. Variables with *p*‐values < 0.1 were included in the adjusted model. A multivariable binary logistic regression model was employed in identifying independent predictors of ITN possession and use. Variables with *p* < 0.1 in bivariate analysis were considered for entry into the multivariable models. Backward stepwise elimination (likelihood‐ratio criterion) was then applied, with variables retained if they remained statistically significant (*p* < 0.05) or were considered a priori confounders based on the literature. Multicollinearity tests were conducted with variance inflation factor scores of 5 and above indicative of collinearity. Multicollinear variables were omitted from the final model. Model fitness was assessed using the Hosmer‐Lemeshow test (*p* > 0.05). All variables in the final multivariable models had VIF values below 5.0, indicating no significant multicollinearity. For the ITN possession model, the Hosmer‐Lemeshow test yielded *χ*² = 6.24, *df* = 8, *p* = 0.621, indicating good model fit. For the ITN utilization model, the test yielded *χ*² = 5.18, *df* = 8, *p* = 0.738, also indicating acceptable model fit.

Results from the multivariable model are presented as adjusted odds ratios (AOR) with their corresponding 95% confidence intervals (CI). The final model was two‐tailed and a *p*‐value of ≤ 0.05 was considered statistically significant.

However, the sample size for the analysis of ITN utilization (*n* = 87) was small, which may limit the precision of the odds ratio estimates and the generalizability of these specific findings. The reasons are that utilization analysis was restricted to ITN owners, explaining the reduced sample. To mitigate this the study employed parsimonious multivariable models to reduce overfitting. Hence, interpretation of findings was done cautiously to avoid causal language.

## 3 Results

### 3.1 General Characteristics of Respondents

Table 1 summarizes the background characteristics of the 159 Fulani pregnant women included in the study. More than half of the respondents were aged 25 years or older (53.5%), with a mean (SD) age of 26.2 (7.1) years. All participants reported Islam as their religion. Nearly half were housewives (47.2%), and the majority had no formal education (78.0%).

**Table 1:** Bivariable analysis of background characteristics of Fulani pregnant women.

Variable	Categorization	*N* (%)	ITN possession (*N* = 159)			ITN utilization (*N* = 87)		
			No (%)	Yes (%)	*p*‐Value	No (%)	Yes (%)	*p* value
Age	< 25	74 (46.5)	37 (50.0)	37 (50.0)	0.338	15 (40.5)	22 (59.5)	0.385
Mean (SD) = 26.18 (7.08)	≥ 25	85 (53.5)	35 (41.2)	50 (58.8)		26 (52.0)	24 (48.0)	
Occupation	Housewife	75 (47.2)	39 (52.0)	36 (48.0)	0.019	22 (61.1)	14 (38.9)	0.077
	Cattle herder/animal rarer/farmer	45 (28.3)	23 (51.1)	22 (48.9)		7 (31.8)	15 (68.2)	
	Trader/vendor/business woman	39 (24.5)	10 (25.6)	29 (74.4)		12 (41.4)	17 (58.6)	
Religion	Islam	159 (100.0)						
Educational status	No formal education	124 (78.0)	54 (43.5)	70 (56.5)	0.446	32 (45.7)	38 (54.3)	0.787
	Formal education	35 (22.0)	18 (51.4)	17 (48.6)		9 (52.9)	8 (47.1)	
Wealth status	Poor	61 (38.4)	19 (31.1)	42 (68.9)	0.006	21 (50.0)	21 (50.0)	0.852
	Medium	34 (21.4)	15 (44.1)	19 (55.9)		8 (42.1)	11 (57.9)	
	Wealthy	64 (40.3)	38 (59.4)	26 (40.6)		12 (46.2)	14 (53.8)	
Household size	≤ 5	96 (60.4)	39 (40.6)	57 (59.4)	0.192	26 (45.6)	31 (54.4)	0.822
Median (range) = 5 (2–9)	> 5	63 (39.6)	33 (52.4)	30 (47.6)		15 (50.0)	15 (50.0)	
Number of children	≤ 3	118 (74.2)	62 (52.5)	56 (47.5)	0.002	36 (64.3)	20 (35.7)	< 0.001
Median (range) = 2 (0–6)	> 3	41 (25.8)	10 (24.4)	31 (75.6)		5 (16.1)	26 (83.9)	
Number of pregnancies	≤ 3	93 (58.5)	52 (55.9)	41 (44.1)	0.002	25 (61.0)	16 (39.0)	0.019
Median (range) = 3 (1–7)	> 3	66 (41.5)	20 (30.3)	46 (69.7)		16 (34.8)	30 (65.2)	
Number of ANC attendance	≤ 3	130 (81.8)	63 (48.5)	67 (51.5)		33 (49.3)	34 (50.7)	
Median (range) = 2 (1–4)	> 3	29 (18.2)	9 (31.0)	20 (69.0)	0.066	8 (40.0)	12 (60.0)	0.611

Abbreviations: ANC, antenatal care; ITN, insecticide‐treated net; SD, standard deviation.

In terms of household socioeconomic status, most women belonged to the poor or medium wealth categories (59.8%). About two in five respondents lived in households with more than five members (39.6%). Regarding reproductive history, 41.5% reported more than three pregnancies, while 25.8% had more than three children. Antenatal care attendance was generally low, with only 18.2% reporting more than three ANC visits during the current pregnancy. Overall, 54.7% of respondents reported possessing an insecticide‐treated bed net, while 45.3% did not (Table 1).

Among women who did not possess an ITN, a higher proportion were younger than 25 years (51.4%) and identified as housewives (54.2%). Most had no formal education (75.0%). More than half of nonowners belonged to the wealthy household category (52.8%), and a similar proportion lived in households with five or fewer members (54.2%). Patterns related to reproductive history and healthcare use also varied. Most women without ITNs reported three or fewer antenatal care visits (87.5%), three or fewer children (86.1%), and three or fewer pregnancies (72.2%) (Table 1).

### 3.2 Bivariable Analysis of Bed Net Possession and Independent Variables

Occupation was associated with ITN possession (*p* = 0.019), with the lowest proportion observed among housewives (48.0%) and the highest among traders, vendors, and businesswomen (74.4%). Wealth status also differed significantly (*p* = 0.006). A higher proportion of women in the poor wealth category possessed ITNs compared with those in the medium and wealthy categories (68.9% vs. 55.9% vs. 40.6%). Reproductive history was similarly associated with ITN possession. A greater proportion of women with more than three children owned ITNs compared with those who had three or fewer children (75.6% vs. 47.5%, *p* = 0.002). Likewise, ITN possession was more common among women with more than three pregnancies than among those with fewer pregnancies (69.7% vs. 44.1%, *p* = 0.002) (Table 1).

However, a higher proportion of women who had ever received counseling on ITNs owned a net compared with those who had not (*p* < 0.001). Significant differences were also observed by source of ITN knowledge (*p* = 0.045), having ever heard of malaria (*p* = 0.018), and reporting a family history of malaria in the previous year (*p* = 0.032). Also, a higher proportion of women who knew that ITNs prevent malaria owned nets compared with those without this knowledge (*p* < 0.001). Similarly, possession was more common among women who correctly identified malaria causes (64.4% vs. 37.9%, *p* = 0.002) and symptoms (63.3% vs. 41.0%, *p* = 0.009). Awareness of other malaria prevention measures was also associated with higher ITN possession (71.7% vs. 47.8%, *p* = 0.008). Overall, women classified as having good malaria knowledge showed a higher proportion of ITN ownership compared with those with poor knowledge (64.9% vs. 38.7%, *p* = 0.002). All women who demonstrated good malaria‐prevention practices reported owning an ITN, compared with 36.3% among those with poorer practices (*p* < 0.001) (Table 2).

**Table 2:** Bivariable analysis of knowledge and practice variables associated with ITN possession and use.

Variable	Categorization	*N* (%)	ITN possession (*N* = 159)			ITN utilization (*N* = 87)		
			No (%)	Yes (%)	*p* value	No (%)	Yes (%)	*p* value
ITN possession	No	72 (45.3)						
	Yes	87 (54.7)						
Number of possessed ITNS	≤ 1	122 (76.7)	72 (59.0)	50 (41.0)	< 0.001^a^	31 (62.0)	19 (38.0)	0.002
Mean (SD) = 0.81 (0.87)	> 1	37 (23.3)	0 (0.0)	37 (100.0)		10 (27.0)	27 (73.0)	
ITN used the previous night	No	41 (25.8)						
	Yes	46 (28.9)						
Frequency of ITN use per week	≤ 3	60 (37.7)				21 (35.0)	39 (65.0)	0.001
	> 3	27 (17.0)				20 (74.1)	7 (25.9)	
Ever counseled on ITN	No	65 (40.9)	42 (64.6)	23 (35.4)	< 0.001	16 (69.6)	7 (30.4)	0.015
	Yes	94 (59.1)	30 (31.9)	64 (68.1)		25 (39.1)	39 (60.9)	
Source of knowledge on ITN	Family/peers/radio	36 (22.6)	16 (44.4)	20 (55.6)	0.045	10 (50.0)	10 (50.0)	0.275
	Health facility	58 (36.5)	14 (24.1)	44 (75.9)		15 (34.1)	29 (65.9)	
Ever heard of malaria	No	42 (26.4)	26 (61.9)	16 (38.1)	0.018	9 (56.3)	7 (43.8)	0.580
	Yes	117 (73.6)	46 (39.3)	71 (60.7)		32 (45.1)	39 (54.9)	
Source of knowledge on malaria	Family/peers/radio	35 (22.0)	15 (42.9)	20 (57.1)	0.681	16 (80.0)	4 (20.0)	< 0.001^a^
	Health facility	82 (51.6)	31 (37.8)	51 (62.2)		16 (31.4)	35 (68.6	
Family member ever had malaria within the year	No	58 (36.5)	33 (56.9)	25 (43.1)	0.032	11 (44.0)	14 (56.0)	0.814
	Yes	101 (63.5)	39 (38.6)	62 (61.4)		30 (48.4)	32 (51.6)	
Knowledge on consequence of malaria	No	30 (18.9)	15 (50.0)	15 (50.0)	0.685	7 (46.7)	8 (53.3)	1.000
	Yes	129 (81.1)	57 (44.2)	72 (55.8)		34 (47.2)	38 (52.8)	
Knowledge that ITN prevents malaria	No	80 (50.3)	49 (61.3)	31 (38.8)	< 0.001	24 (77.4)	7 (22.6)	< 0.001
	Yes	79 (49.7)	23 (29.1)	56 (70.9)		17 (30.4)	39 (69.6)	
Knowledge on cause of malaria	No	58 (36.5)	36 (62.1)	22 (37.9)	0.002	15 (68.2)	7 (31.8)	0.027
	Yes	101 (63.5)	36 (35.6)	65 (64.4)		26 (40.0)	39 (60.0)	
Knowledge on signs and symptoms of malaria	No	61 (38.4)	36 (59.0)	25 (41.0)	0.009	18 (72.0)	7 (28.0)	0.004
	Yes	98 (61.6)	36 (36.7)	62 (63.3)		23 (37.1)	39 (62.9)	
Knowledge on malaria preventive measures aside ITN	No	113 (71.1)	59 (52.2)	54 (47.8)	0.008	41 (75.9)	13 (24.1)	< 0.001
	Yes	46 (28.9)	13 (28.3)	33 (71.7)		0 (0.0)	33 (100.0)	
Knowledge level on malaria	Poor	62 (39.0)	38 (61.3)	24 (38.7)	0.002	17 (70.8)	7 (29.2)	0.008^a^
Mean (SD) = 3.58 (2.01), Median (IQR) = 4 (1–5)	Good	97 (61.0)	34 (35.1)	63 (64.9)		24 (38.1)	39 (61.9)	
Practices towards malaria prevention	Poor	113 (71.1)	72 (63.7)	41 (36.3)	< 0.001^a^	41 (100.0)	0 (0.0)	< 0.001^a^
	Good	46 (28.9)	0 (0.0)	46 (100.0)		0 (0.0)	46 (100.0)	

Abbreviations: IQR, interquartile range; ITN, insecticide‐treated net; SD, standard deviation.

^a^ All test were from Fisher's exact tests.

**Figure 2:**
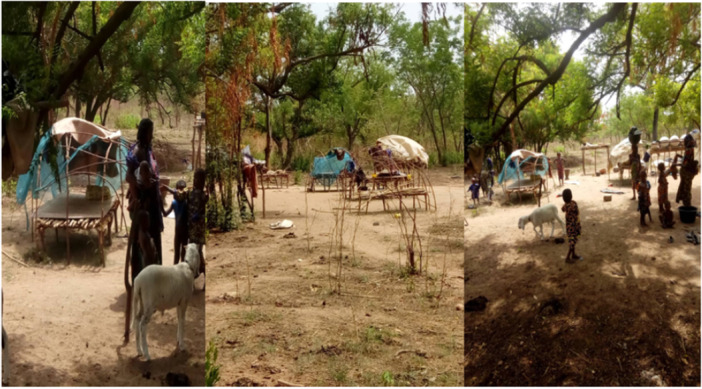
Images of Fulani pregnant women's homes surrounded with mosquito bed net. *Source:* Field Survey 2022, West Gonja Municipality.

A pictorial representation of Fulani households surrounded by mosquito bed nets is shown in Figure 2.

### 3.3 Bivariable Analysis of Bed Net Utilization and Independent Variables

Among the 159 Fulani pregnant women surveyed, 54.7% reported owning at least one ITN. However, only 28.9% of the total sample reported sleeping under a net the night before the survey. Among ITN owners, over half (52.8%) reported net use the previous night (Table 2).

Also, a higher proportion of women with more than three children reported using an ITN compared with those with three or fewer children (83.9% vs. 35.7%, *p* < 0.001). Similarly, ITN use was more common among women with more than three pregnancies than among those with fewer pregnancies (65.2% vs. 39.0%, *p* = 0.019) (Table 1).

Additionally, a higher proportion of women who owned multiple ITNs reported sleeping under a net compared with those who owned only one (*p* = 0.002). ITN use was also more common among women who had ever received counseling on ITNs (*p* = 0.015), those with different sources of malaria‐related information (*p* < 0.001), and those who knew that ITNs prevent malaria (*p* < 0.001) (Table 2).

Correct knowledge of malaria causes (60.0% vs. 31.8%, *p* = 0.027) and symptoms (62.9% vs. 28.0%, *p* = 0.004) was associated with higher proportions of ITN use. Women who were aware of additional malaria prevention measures reported substantially higher ITN use compared with those who were not (100.0% vs. 24.1%, *p* < 0.001). Overall, ITN use was more common among women with good malaria knowledge (61.9% vs. 29.2%, *p* = 0.008) and among those demonstrating positive malaria‐prevention practices (*p* < 0.001) (Table 2).

### 3.4 Multivariable Analysis

#### 3.4.1 Factors Associated With ITN Possession

After controlling for confounding variables in the multivariable analysis, three key predictors of ITN possession were identified: knowledge that ITN prevents malaria, ever counseled on ITN and wealth status of respondents. Individuals who had knowledge that ITN prevents malaria had 3.45 times higher odds of bed net possession as compared to those who did not (AOR = 3.45, 95%[CI] = 1.333–8.935, *p* = 0.011) (Table 3). Respondents who had ever received counseling on ITN were about 4.52 times higher odds of possessing ITN over their counterparts (AOR = 4.517, 95%[CI] = 1.529–13.339, *p* = 0.006). Fulani pregnant women who possessed a higher wealth index (wealthy status) had 0.21 lower odds of bed net possession as compared to their peers in the other groups (AOR = 0.211, 95%[CI] = 0.091–0.491, *p* < 0.001). As shown in Table 3, general knowledge on malaria was not associated with ITN possession.

**Table 3:** Multivariable analysis of factors associated with ITN possession among respondents.

Variable	AOR (95%CI)	*p* value
Knowledge that ITN prevents malaria: No (Ref) vs. Yes	3.451 (1.333–8.935)	0.011
Ever counseled on ITN: No (Ref) vs. Yes	4.517 (1.529–13.339)	0.006
Knowledge level on malaria: Poor (Ref) vs. Good	0.489 (0.142–1.680)	0.256
Wealth status: Poor (Ref)		
Medium	0.466 (0.175–1.241)	0.127
Wealthy	0.211 (0.091–0.491)	< 0.001

Abbreviations: AOR, adjusted odds ratio; CI, confidence interval; ITN, insecticide‐treated net.

### 3.5 Factors Associated With ITN Utilization

Various factors that influence ITN utilization were evaluated using multivariable analysis. Identifiable factors significantly related to ITN use in the current study were number of children, knowledge on signs and symptoms of malaria and number of ITN possessed. As highlighted in Table 4, those who had ≤ 3 children were 98.5% lower odds to use ITNs than their peers (AOR = 0.015, 95%[CI] = 0.002–0.123, *p* < 0.001). Respondents who possessed ≤ 1 ITNs were 93.1% lower odds to use ITNS than their counterparts (AOR = 0.069, 95%[CI] = 0.017–0.282, *p* < 0.001). Fulani pregnant women who possessed no knowledge on the signs and symptoms of malaria were 82.5% lower odds to use ITNs that their colleagues (AOR = 0.175, 95%[CI] = 0.048–0.636, *p* = 0.008) (Table 4).

**Table 4:** Multivariable analysis of factors associated with ITN utilization among Fulani pregnant women.

Variable	AOR (95%CI)	*p* value
Number of pregnancies: ≤ 3 vs. > 3 (Ref)	2.570 (0.520–12.703)	0.247
Number of children: ≤ 3 vs. > 3 (Ref)	0.015 (0.002–0.123)	< 0.001
Number of possessed ITNs: ≤ 1 vs. > 1 (Ref)	0.069 (0.017–0.282)	< 0.001
Knowledge on signs and symptoms of malaria: No vs. Yes (Ref)	0.175 (0.048–0.636)	0.008

Abbreviations: AOR, adjusted odds ratio; CI, confidence interval; ITN, insecticide‐treated net.

## 4 Discussion

Insecticide‐treated nets remain one of the most cost‐effective malaria prevention tools in sub‐Saharan Africa. Yet, consistent with findings across the region, coverage and use often fall short of recommended targets, especially among high‐risk groups such as pregnant women [26, 27]. In this study, just over half of Fulani pregnant women reported owning an ITN (54.7%) and using it the previous night (52.8%). These rates are substantially lower than national or mixed‐population estimates in Sierra Leone (87.6% possession; 76.5% use) [28] and other African countries, where ownership ranged from 63.5% to 98.9% and usage from 55.9% to 87.1% [26, 27, 29, 30, 31, 32, 33, 34, 35, 36]. However, they are more comparable to the low levels observed among pregnant women in Nigeria (28.8% possession; 25.7% use) [37]. The lower ITN ownership and utilization observed in this study compared with national and mixed population estimates likely reflects the intersecting effects of nomadic mobility, temporary housing structures, outdoor sleeping practices, and limited engagement with routine antenatal and mass distribution platforms. Among Fulani households, these structural and cultural factors constrain both access to ITNs and their consistent nightly use, even when knowledge is present. This context‐specific interaction helps explain discrepancies between our findings and those from more sedentary populations.

A central finding of this study is that ITN ownership did not guarantee use. Only half of those who owned nets reported sleeping under them. This reflects patterns reported elsewhere, where discomfort (heat, odor, and space constraints), misperceptions, and poor handling discouraged consistent use [38, 39, 40, 41]. On the other hand, knowledge and counseling were strongly associated with higher possession and use. Women who knew that ITNs prevent malaria had more than threefold greater odds of ownership, while those counseled during antenatal care were over four times more likely to possess nets. These findings are consistent with evidence from Nigeria and other African contexts [37, 38, 42, 43], and they reinforce health behavior models which suggest that protective practices are sustained only when awareness and motivation accompany material access [44].

Socioeconomic differences also shaped ITN patterns in unexpected ways. Women from wealthier households were less likely to own nets, a wealth paradox also described in previous studies [29, 45]. This may reflect reliance on alternative preventive measures such as insecticide sprays or repellents, or residence in better‐constructed houses with fewer mosquito entry points. Conversely, poorer women, who face greater economic risks from malaria treatment may be more inclined to use nets when available. Household factors further influenced uptake: families with multiple ITNs were more likely to report consistent use, while smaller households (≤ 3 children) were less likely to do so. Similar associations have been reported in Ethiopia, Chad, Nigeria, and Uganda [29, 30, 36, 46], suggesting that both household composition and resource distribution are critical in shaping ITN practices.

The Fulani context provides unique insights that extend the broader literature. Their nomadic lifestyle, geographic isolation, and limited integration into mainstream health programs reduce opportunities to benefit from ITN distributions tied to antenatal care, community campaigns, or seasonal malaria chemoprevention [45, 47]. This helps explain why coverage in this study falls below WHO's benchmark of ≥ 80% for household ownership and use [48]. Addressing these gaps requires interventions tailored to nomadic and marginalized populations. Practical approaches could include mobile health units, community‐driven distribution systems, and culturally adapted counseling in local languages. Equally, strategies should account for wealth‐related differences, framing ITNs not as the sole preventive option but as a reliable and complementary measure alongside other tools.

### 4.1 Limitations and Strengths

This study was conducted during the late dry season to early rainy season, which may have influenced ITN use, as seasonal variation affects mosquito density and perceived malaria risk. Several potential biases should be acknowledged. Social desirability bias may have led to overreporting of ITN use, although anonymity was emphasized to reduce this effect. The snowball sampling approach introduced possible gatekeeper and selection biases, as initial “seed” participants could have influenced the composition of the sample. Excluding three communities due to inaccessibility may have further underrepresented remote Fulani groups. The small sample size and cross‐sectional design also limit generalizability and prevent cause–effect interpretations. Furthermore, the sample size for the analysis of ITN utilization (*n* = 87) was small, which may limit the precision of the odds ratio estimates and the generalizability of these specific findings. Additionally, some determinants such as attitudes and practices toward ITNs were not explored.

Given that data collection occurred during the transition from late dry season to early rainy season, ITN use patterns may differ from those observed during peak transmission periods. Future studies should consider longitudinal designs that capture seasonal variations in both ITN possession and utilization to better understand how malaria risk perception and preventive behaviors fluctuate throughout the year. Such evidence would inform more targeted and timely distribution strategies tailored to nomadic populations. Future studies should employ longitudinal designs that account for seasonal variability in malaria transmission and ITN use, particularly among mobile pastoralist communities whose movement patterns may also follow seasonal rhythms.

Despite these limitations, the study has important strengths. It is the first to examine ITN possession and use among Fulani nomadic pregnant women in Ghana, providing rare insight into a marginalized and highly mobile population. The recruitment approach was effective, as trust‐building was critical for engaging this community. Findings add to the limited body of evidence on health among nomadic groups and can inform culturally tailored malaria prevention policies and programs. Future research with larger, longitudinal samples is needed to confirm these results and guide interventions targeting mobile and underserved populations.

## 5 Conclusions

This study shows that ownership and use of insecticide‐treated nets among nomadic Fulani pregnant women in Ghana's savannah zone remain unacceptably low, with outcomes strongly influenced by education, occupation, household wealth, antenatal care attendance, prior counseling and malaria knowledge.

Therefore, interventions must be adapted to the unique realities of nomadic populations. Mobile distribution linked to antenatal outreach can expand coverage among women with limited access to health facilities. Moreover, health promotion delivered in local languages through community and religious leaders can improve knowledge and address cultural misconceptions. Additionally, economic barriers can be reduced through subsidies or non‐governmental organization (NGO)‐supported free provision, while school and mosque‐based programs can further strengthen long‐term awareness and uptake.

However, effective implementation requires coordinated action across stakeholders. Moreover, community leaders should mobilize trust and acceptance, while health providers need to enhance ITN counseling embedded into routine antenatal care. Also, NGOs should extend tailored outreach to remote areas, and government agencies should guarantee consistent ITN supply and align policies with national malaria control strategies.

In addition, qualitative research is essential to explore household power dynamics, particularly the influence of men in decision‐making about ITN acquisition and use. In doing so, evidence from such studies will inform culturally grounded strategies that are more responsive to community needs. Future studies should employ longitudinal designs and larger samples to confirm these associations and explore underlying mechanisms, such as intra‐household decision‐making and the specific logistical barriers faced by mobile pastoralist communities. Nonetheless, integrating these actions, policymakers and partners can advance Sustainable Development Goal 3 on good health and well‐being, reduce malaria‐related risks during pregnancy, and promote equity in maternal and child health among nomadic populations.

## Author Contributions

**Yula Salifu:** conceptualization, investigation, writing – original draft, methodology, validation, visualization, writing – review and editing, software, formal analysis, project administration, data curation, supervision, resources. **Joseph Lasong:** conceptualization, investigation, funding acquisition, writing – original draft, methodology, validation, visualization, writing – review and editing, project administration, data curation, supervision, resources. **Eleonora Bakintewune Wobi:** methodology, validation, visualization, writing – review and editing, funding acquisition, project administration. **Torjim Salifu:** methodology, validation, visualization, writing – review and editing, software, formal analysis, project administration, data curation. **Bismark Nantomah:** writing – original draft, data curation, supervision, resources, project administration, writing – review and editing. **Gordon Dandeebo:** writing – review and editing, visualization, validation, methodology, project administration, supervision.

## Funding

The authors have nothing to report.

## Ethics Statement

Respondents made aware of the study's purpose. In the questionnaire administration, concern and consent were sought from respondents. Contribution to this research was optional. Study permission and approval was acquired from the West Gonja Municipal Health Directorate and the Institutional Review Board of the University for Development Studies with reference ID: [UDS/RB/059/22] respectively.

## Consent

The authors have nothing to report.

## Conflicts of Interest

The authors declare no conflicts of interest.

## Transparency Statement

The lead author Joseph Lasong affirms that this manuscript is an honest, accurate, and transparent account of the study being reported; that no important aspects of the study have been omitted; and that any discrepancies from the study as planned (and, if relevant, registered) have been explained.

## Supporting information

Supporting File

## Data Availability

The datasets generated and analyzed during the current study are not publicly available due to privacy and ethical restrictions concerning this vulnerable population but are available from the corresponding author on reasonable request.
