# Supplementary material for: Factors Associated With Insecticide‐Treated Bed Net Possession and Utilization in Malaria Prevention Among Fulani Pregnant Women in the Savannah Hinterlands of Ghana: A Cross‐Sectional Study
Source: Health Sci Rep. 2026 Apr 20;9(4):e72415. doi: 10.1002/hsr2.72415 (PMC13096573; doi:10.1002/hsr2.72415)
Supplement: Supplementary file 1 — Supporting File [file HSR2-9-e72415-s001.docx]

**Questionnaires**

**Possession, Utilization, Knowledge and Practice Questionnaire about Malaria and Its Preventive Measures among Nomadic Fulani pregnant women in the West Gonja Municipality of the Savannah Region of Ghana**

**Dear Participant,**

This survey is in the process of improving the awareness, attitude and behavior of Fulani pregnant women on malaria and its preventive options, so that adequate knowledge about your concerns will be considered in order to better serve and address all your malaria knowledge and practice towards its prevention issues. The questionnaire is divided into **2 Sections** including: **Basic information of participant and Knowledge and practices of participants towards malaria prevention.** The questionnaire is completely anonymous and please your sincerity and input are very crucial in helping us attain our goal.

**Questionnaire serial No (Anonymous survey – Please do not fill the blank space) [___/___/___/___]**

Do you agree to participate in the survey: □Yes □No?

**SECTION A**

**(SOCIO-DEMOGRAPHIC CHARACTERISTICS OF PARTICIPANTS)**

**Please tick/fill any option that applies**

1. COMMUNITY NAME/GEOGRAPHICAL LOCATION

……………………………………………………

1. How old are you (years)?................................................
2. What is your marital status?
3. Never Married
4. Married/Cohabiting
5. Divorced/Widowed
6. If married/cohabiting, how many wives does your husband have?
7. 1
8. 2
9. 3
10. Others please specify?...............................................
11. What is your educational status?
12. No formal education
13. Primary
14. J.H.S
15. S.H. S
16. Tertiary
17. What is your religion?
18. Islam
19. Christianity
20. Others, please specify?……………………………
21. What is your occupation?
22. Cattle-Herder/Animal rarer/ Farmer
23. Trader/Vendor/Business woman
24. Housewife
25. Others, please specify?……………………………
26. How many people do you live with and eat from the same pot (e.g., husband, children, grandparents)
27. 1
28. 2
29. 3
30. Others, please specify?……………………………
31. What is your parity status?
32. 0
33. 1
34. 2
35. Others, please specify?..............................…….
36. What is your gravidity status?
37. 1
38. 2
39. Others, please specify?……………………………………
40. Have you ever attended ANC during this current pregnancy?
41. Yes
42. No
43. If yes, how many times?…………………………………..
44. What is your current estimated gestational age in weeks? ……………………..
45. How old is your current child, if any/ what is the spacing between your current child and this pregnancy in years?……………………………………….

**Household Wealth Status Assessment Indicators**

**Please tick all/any that applies**

1. Do you possess any of the following **household assets**?
2. Motorcycle/bike
3. Bicycle
4. Motor king/Tricycle
5. Radio/Stereo
6. Mattress/Bed/Sleeping Mat
7. Torchlight/Lamp/Lantern/Bulb
8. Electric/Solar powered Fan
9. Mobile phone
10. Computer
11. DVD/VCD Player
12. Animal-drawn Cart
13. Speaker
14. Television
15. Gas cooker/coal pot/stove (mud/clay/stone)
16. What source of energy do you use to cook?
17. Charcoal/Firewood
18. Electricity/LP Gas
19. Biogas
20. What source of lighting do you use in your house?
21. Electricity/solar
22. biogas
23. Battery
24. Do you have a toilet facility?
25. Yes
26. No
27. If yes, what type of toilet facility do you use?
28. KVIP
29. Pit latrine
30. Flushing toilet
31. Open defecation
32. What housing structure do you possess?
33. Mud
34. Concrete
35. Part mud, part concrete
36. What kind of roofing structure do you possess?
37. Aluminum/Alu-zinc
38. Thatch/Hay/Straw
39. What is your source of drinking water?
40. Bore hole
41. Tap/Pipe borne water
42. Well, /Spring
43. Dam/River/Stream/Pond

**SECTION B**

**(KNOWLEDGE AND PRACTICES OF PARTICIPANTS TOWARDS MALARIA PREVENTION)**

**Please tick/fill option(s) that apply**

1. Have you heard of Malaria before?
2. Yes
3. No
4. If yes, where/who did you receive the information on malaria from?
5. Family members
6. Peers/Friends
7. Radio
8. Health facility
9. Others, please specify?……………………………..…
10. Who is most at-risk of contracting malaria?
11. Children below 5-years
12. Any person
13. Pregnant women
14. Aged persons
15. Others, please specify?……………………………
16. Have you ever had Malaria during this current pregnancy?
17. Yes
18. No
19. Has any of your family members ever had malaria within the past 12 months?
20. Yes
21. No
22. Do you possess an insecticide treated mosquito bed net(s)?
23. Yes
24. No
25. If yes, how many insecticides treated mosquito bed net(s) do you have?
26. 1
27. 2
28. 3
29. Others, please specify?……………………………
30. Did you use the insecticide treated mosquito bed net(s) a night before this survey?
31. Yes
32. No
33. Do you frequently sleep under the insecticide treated mosquito bed net(s)?
34. Yes
35. No
36. If yes how many times within a week do you sleep under the insecticide treated mosquito bed net(s)?
37. 1
38. 2
39. 3
40. Others, please specify?……………………………
41. Have you ever received counselling on insecticide treated mosquito bed net(s) possession and/or usage?
42. Yes
43. No
44. If yes, where/who did you receive the counselling on insecticide treated mosquito bed net(s) possession and/or usage from?
45. Family members
46. Peers/Friends
47. Radio
48. Health facility
49. Others, please specify?……………………………
50. Is malaria risky to both mother and fetus during pregnancy?
51. Yes
52. No
53. If yes, what are the consequences of malaria for both mother and fetus?
54. Miscarriage
55. Congenital malformations
56. Anemia
57. Others please specify?....................................
58. Do insecticide treated bed net(s) prevent malaria?
59. Yes
60. No
61. Is insecticide treated bed net(s) the only malaria prevent method?
62. Yes
63. No
64. If no, what other malaria preventive methods do you know?
65. Herbal remedies
66. Spirituality/Divine protection
67. Indoor residual spraying
68. Mosquito coils
69. Others please specify?..................................................
70. Do mosquito bites lead to malaria in pregnancy?
71. Yes
72. No
73. When do mosquito bites occur?
74. During the day
75. During the night
76. At any time
77. What signs and symptoms of malaria of malaria do you know?
78. Fever
79. Headaches
80. Loss of appetite
81. Do not know
82. Others please specify?................................
83. Do you consume IPTp-SP during your current pregnancy?
84. Yes
85. No
86. If yes, how many have you consumed during your current pregnancy?
87. 1
88. 2
89. 3
90. Others please specify?................................................
91. Have you consumed any medication aside IPTp-SP during your current pregnancy to prevent malaria?
92. Yes
93. No
94. Have you received any visits for Seasonal Malaria Chemoprevention (SMC) during your current pregnancy?
95. Yes
96. No
97. If yes, how many times have you benefited from the SMC program?
98. 1
99. 2
100. 3
101. Others please specify?................................................

THANK YOU FOR YOUR RESPONSE

Interview time: ……………. Interview location: ……….………Recorder.…………

Investigator ID and signature: ______________ Date of survey: _______________

Check and verified by (ID and signature): ________Date of verification: _______

____________________________________________________________________________________________
